# Breast Cancer Relapse, Post-Surgical Confusion, and Dementia in the Elderly: An Unexpected Connection but with the Same Proposed Solution

**DOI:** 10.3390/medicina54060101

**Published:** 2018-12-03

**Authors:** Michael W. Retsky, Patrice Forget

**Affiliations:** 1 Department of Environmental Health, Harvard TH Chan School of Public Health, Boston, MA 02115, USA, mretsky@hsph.harvard.edu; 2 Anesthesiology and Perioperative Medicine, Universitair Ziekenhuis Brussel, Vrije Universiteit Brussel, 1090 Brussels, Belgium

**Keywords:** breast cancer relapse, confusion, dementia, inflammation

## Abstract

A simple solution may exist for both the problem of sudden dementia and confusion after surgery in the elderly and the bimodal relapse pattern among breast cancer patients who were treated with a mastectomy. Systemic inflammation by a variety of mechanisms can induce tumor outgrowth from dormant states, such as single dormant cells and avascular micrometastases. This may also explain sudden confusion and dementia for the elderly after surgery. We propose that surgery-induced inflammation may be addressed by “protective anesthesia”. We suggest ketorolac for 4 days starting at the time of surgery to prevent early relapse in breast and probably other cancers; perhaps that or something similar could be used before surgery in elderly patients to prevent post-operative cognitive dysfunction.

Dear Editor,

We have been actively conducting research in breast cancer and have completed an extensive paper trail; thus, details need not be presented in this short communication [1]. The interesting phenomenon we want to highlight is that our research program has led us to propose what may be a simple solution to the problem of sudden dementia and confusion after surgery in the elderly. This has long been assumed to be the result of general anesthesia but is still unresolved.

In brief, we have been studying a bimodal relapse pattern among breast cancer patients who were treated with mastectomy only. An analysis of these data using computer simulation suggested that relapses within three years of primary removal were stimulated from dormant phases into active growth that correlated with the time of surgery. A plausible explanation for this observation resulted when a retrospective study from Brussels reported that use of the perioperative NSAID ketorolac reduced early relapses 5-fold.

Combining our resources, we concluded that primary surgery results in a period of approximately one week during which systemic inflammation is induced. In support of this, an increase in the inflammatory marker IL-6 has been identified in serum post-surgery. Systemic inflammation by a variety of mechanisms can induce tumor outgrowth from dormant states such as single dormant cells and avascular micrometastases. Relapses in the first three post-surgery years are the apparent result. We have published a book on this research in 2017 by Nature/Springer that entails an extensive review.

Systemic inflammation occurs after many types of provocation and can cause a number of effects that show up in different clinical settings. These include post-surgical peripheral neuropathy [2], Guillain-Barre-syndrome [3], death from heart failure after hip fracture [4], death from new cancers and also from cardiac events associated with external beam radiation to prevent local relapse [5]. We now suspect this may also explain sudden confusion and dementia for the elderly after surgery, which has been blamed on general anesthesia.

According to Hussain et al. [6], the effect of surgery in aged mice shows cognitive dysfunction that seems to result from neuroinflammation. Surgery leads to the production of tumor necrosis factor α (TNF-α), which subsequently disrupts the blood-brain barrier, causing an infiltration of inflammatory macrophages in the brain parenchyma, specifically the hippocampus. The levels of the inflammatory cytokines IL-1β and IL-6 have been shown to increase in mice that underwent surgery, compared to mice that only received anesthesia. The levels of cytokines in the group that received anesthesia without surgery were comparable to anesthesia-naïve control mice. They reported that the inflammatory response and resultant cognitive deficits in mice might largely be a result of surgery rather than due to any specific effects of general anesthesia.

There are similar human data. Kudoh et al. [7] considered 80 patients aged 70–90 years who underwent abdominal surgery and measured plasma IL-6, cortisol or noradrenaline concentrations before and after surgery. They found a positive correlation between IL-6 and cortisol after surgery and the development of postoperative confusion. This is consistent with the observation that sedation after cardiac surgery with dexmedetomidine, a medication known to decrease adrenergic stress with promising anti-inflammatory action in animal models, decreases the incidence of delirium [8,9].

Rather than general anesthesia having a presumptive deleterious effect on cognition, we propose that surgery-induced inflammation could be the main driver of cognitive impairment and confusion in elderly persons after surgery. The goal should be to find ‘protective anesthesia’. We have suggested ketorolac for 4 days starting at the time of surgery to prevent early relapse in breast and probably other cancers; perhaps that or something similar could be used before surgery in elderly patients to prevent post-operative cognitive dysfunction. Nevertheless, the research agenda still comprises the demonstration that the reduction of systemic inflammation is possible in humans, to a level that is beneficial, and then to prevent the aforementioned conditions.
